# Luteal Support with very Low Daily Dose of Human Chorionic Gonadotropin after Fresh Embryo Transfer as an Alternative to Cycle Segmentation for High Responders Patients Undergoing Gonadotropin-Releasing Hormone Agonist-Triggered IVF

**DOI:** 10.3390/ph14030228

**Published:** 2021-03-07

**Authors:** Andrea Roberto Carosso, Stefano Canosa, Gianluca Gennarelli, Marta Sestero, Bernadette Evangelisti, Lorena Charrier, Loredana Bergandi, Chiara Benedetto, Alberto Revelli

**Affiliations:** 1 Obstetrics and Gynecology 1U, Physiopathology of Reproduction and IVF Unit, Department of Surgical Sciences, Sant’Anna Hospital, University of Torino, 10042 Turin, Italy; s.canosa88@gmail.com (S.C.); gennarelligl@gmail.com (G.G.); sesteromarta@gmail.com (M.S.); betty.evangelisti@gmail.com (B.E.); chiara.benedetto@unito.it (C.B.); alberto.revelli@unito.it (A.R.); 2 Department of Public Health and Pediatrics, University of Torino, Via Santena, 5 bis, 10126 Torino, Italy; lorena.charrier@unito.it; 3 Department of Oncology, University of Torino, Via Santena 5 bis, 10126 Torino, Italy; loredana.bergandi@unito.it

**Keywords:** luteal phase support, corpus luteum function, GnRH agonist trigger, hCG, GnRH antagonist protocol, IVF, ovarian hyperstimulation syndrome

## Abstract

The segmentation of the in vitro fertilization (IVF) cycle, consisting of the freezing of all embryos and the postponement of embryo transfer (ET), has become popular in recent years, with the main purpose of preventing ovarian hyperstimulation syndrome (OHSS) in patients with high response to controlled ovarian stimulation (COS). Indeed cycle segmentation (CS), especially when coupled to a GnRH-agonist trigger, was shown to reduce the incidence of OHSS in high-risk patients. However, CS increases the economic costs and the work amount for IVF laboratories. An alternative strategy is to perform a fresh ET in association with intensive luteal phase pharmacological support, able to overcome the negative effects of the GnRH-agonist trigger on the luteal phase and on endometrial receptivity. In order to compare these two strategies, we performed a retrospective, real-life cohort study including 240 non-polycystic ovarian syndrome (PCO) women with expected high responsiveness to COS (AMH >2.5 ng/mL), who received either fresh ET plus 100 IU daily human chorionic gonadotropin (hCG) as luteal support (FRESH group, *n* = 133), or cycle segmentation with freezing of all embryos and postponed ET (CS group, *n* = 107). The primary outcomes were: implantation rate (IR), live birth rate (LBR) after the first ET, and incidence of OHSS. Overall, significantly higher IR and LBR were observed in the CS group than in the FRESH group (42.9% vs. 27.8%, *p* < 0.05 and 32.7% vs. 19.5%, *p* < 0.05, respectively); the superiority of CS strategy was particularly evident when 16–19 oocytes were retrieved (LBR 42.2% vs. 9.5%, *p* = 0.01). Mild OHSS appeared with the same incidence in the two groups, whereas moderate and severe OHSS forms were observed only in the FRESH group (1.5% and 0.8%, respectively). In conclusion, in non-PCO women, high responders submitted to COS with the GnRH-antagonist protocol and GnRH-agonist trigger, CS strategy was associated with higher IR and LBR than the strategy including fresh ET followed by luteal phase support with a low daily hCG dose. CS appears to be advisable, especially when >15 oocytes are retrieved.

## 1. Introduction

In reproductive physiology, the luteinizing hormone (LH) is mandatory for maintaining corpus luteum function and promotes the secretion of progesterone and growth factors involved in embryo implantation and placentation [1,2]. 

In in vitro fertilization (IVF), suppression of hypothalamic activity by GnRH analogs/antagonists is required during controlled ovarian stimulation (COS) of multiple follicles in order to avoid a premature LH surge. Final oocyte maturation is commonly triggered by administering exogenous human chorionic gonadotropin (hCG). Indeed, due to its strong biological activity and its relatively long half-life [3], hCG is able to sustain the multiple corpora lutea throughout the luteal phase. Nevertheless, in order to compensate for the supra-physiological pre-ovulatory levels of estradiol, observed during COS [4,5], the addition of progesterone is needed for optimal endometrial receptivity and to avoid early pregnancy loss [6,7]. 

In women who have a large ovarian reserve (defined as “high responders”), the administration of exogenous hCG in the presence of supraphysiological estradiol levels is associated with an increased risk of ovarian hyperstimulation syndrome (OHSS) [8], partly caused by the stimulating effects of hCG on ovarian production of vasoactive factors [9,10]. 

The risk of OHSS may be minimized using “short protocols” with GnRH antagonists to accomplish COS, and triggering ovulation with a bolus of GnRH-agonist instead of hCG [11,12,13]. With a GnRH-agonist trigger, a physiological peak of LH is responsible for final oocyte maturation. Whereas the number of retrieved mature oocytes is usually comparable after either a GnRH-agonist or hCG trigger [11], the rapid drop of endogenous LH concentration observed after a GnRH-agonist trigger negatively affects corpus luteum function, in turn worsening endometrial receptivity [14]. Indeed, luteal phase deficiency after GnRH-agonist trigger administration is extremely frequent, resulting in lower implantation and clinical pregnancy rates [15], even when progesterone supplementation is used. 

In order to solve this problem, freezing of all embryos and delaying embryo transfer to the following ovarian cycle has been proposed. This strategy is defined as cycle segmentation (CS) or “freeze-all” strategy. With CS, the risk of OHSS is strongly reduced, whereas IVF efficacy is maintained [16]. However, such an approach increases both the economic costs and the amount of work for the IVF unit. Furthermore, some patients consider the delay of embryo transfer (ET) negatively, and their anxiety level increases, enhancing the risk of drop-out.

As alternatives to CS, some forms of intensive pharmacological supplementation of the luteal phase after the GnRH-agonist trigger have been suggested. Among others, the addition of estradiol to progesterone at high doses [17,18], or the administration of 1500 IU hCG the day of oocyte pick up (OPU) [14,19]. Promising results have been obtained with the daily administration of a very low (100 IU) dose of hCG in the luteal phase, which was proposed in a small series of patients who underwent GnRH-agonist trigger and fresh ET [20]. 

The aim of the present study was to compare the outcome of this approach, that is, fresh ET followed by a very low daily dose of hCG, with CS, applied to a group of non-PCO patients treated with a GnRH-analogue trigger, who were expected to be high responders on the basis of their ovarian reserve markers. The primary end-points were the implantation rate (IR), the live birth rate (LBR), and the ovarian hyperstimulation syndrome (OHSS) incidence. 

## 2. Results

The baseline clinical characteristics of the women included in the study are shown in Table 1. Table 2 shows the outcome of IVF in the two study groups.

Women in the CS group had higher ovarian reserve markers (AMH and AFC): although the mean and total dose of exogenous FSH was comparable in the two groups, they finally got a higher number of retrieved oocytes and blastocysts (Table 2). However, the mean embryo score was comparable in the two groups. The higher responsiveness to COS in the CS group did not influence the calculation of the implantation rate (IR), clinical pregnancy rate (CPR), or the live birth rate (LBR), as it was performed taking into account only the first ET, and did not cumulatively consider all ETs. As per the protocol, all viable and transferable blastocysts in the CS group were vitrified, but those after the first ET were not considered to calculate the outcomes. 

Overall, we observed significantly higher IR, CPR and LBR after the first ET in the CS group than in the FRESH group (IR = 42.9% vs. 27.8%, *p* < 0.05; CPR = 40.2% vs. 26.3%, *p* < 0.05; LBR = 32.7% vs. 19.5%, *p* < 0.05, respectively). 

Due to the significant association between the number of retrieved oocytes and the study groups (*p* < 0.01), patients were then stratified according to the number of retrieved oocytes (Table 3; A = 8–11 oocytes, B = 12–15 oocytes, C = 16–19 oocytes) to compare the FRESH and CS groups in three subsets of patients with almost no significant differences in the baseline characteristics and with comparable responsiveness to COS. Even after normalizing for the response to COS, which virtually abolished all effects due to different ovarian reserve markers in the two “naïve” groups, we observed that the CS strategy led to higher, although not statistically significant, percentage of positive hCG test and LBR, and that its superiority was particularly remarkable when >15 oocytes were retrieved (Table 3). These results were confirmed after fitting logistic regression models in the three subgroups of women, where LBR was the dependent variable, and the study group (CS vs. FRESH), AMH and estradiol levels were the independent variables: after adjusting for AMH and estradiol levels, the CS group showed a higher probability of LBR in all subgroups, with the association being nearly significant (OR 4.43, CI95% 0.84; 23.2, *p* = 0.07, data not shown) among women with >15 retrieved oocytes. 

A total number of 29 events of mild OHSS were observed, with no significant difference between the two groups (12% vs. 12.1%). Moderate and severe OHSS were observed with two and one cases in the FRESH group, respectively (1.5% and 0.8%, respectively, Table 3), whereas no cases were reported in the CS group (Table 2).

## 3. Discussion

In recent years, the freezing of all available embryos followed by thaw ET in one of the subsequent spontaneous cycles, namely known as “cycle segmentation”, has become quite popular. The main reason for this is the efficacy in preventing OHSS in high-risk patients, among which are both women with PCO ovaries and those with very marked responsiveness to COS, the so-called “high responders” [21,22,23,24,25]. These patients are usually submitted to a COS protocol, including the use of low-dose gonadotropins plus a GnRH-antagonist to prevent premature LH peak. The trigger of final follicular maturation is obtained using a single bolus of GnRH-agonist, which stimulates the release of an endogenous LH peak. 

In comparison with the classical hCG trigger, the GnRH-agonist-stimulated LH peak remarkably lowers the risk of inducing OHSS, but it is shorter and biologically weaker, resulting in insufficient support for the corpus luteum during the luteal phase [26]. This does not represent an issue when all embryos are vitrified and ET is postponed to one of the following cycles. 

Besides the documented efficacy in preventing OHSS, CS may offer further advantages, such as a more favorable pattern of endometrial gene expression and increased receptivity [26], both linked to the absence of detrimental endometrial effects caused by excessive E2 levels and/or premature P elevation [27]. Indeed, COS has been associated with altered angiogenesis, impaired placentation [28,29,30], and higher instability of the reproductive tract microbiota [31], all factors able to reduce the likelihood of blastocyst implantation. As a matter of fact, in specific subgroups of patients, CS have been reported to be associated with higher implantation rates [21,32] and better perinatal and obstetric outcomes [33,34] than the conventional fresh ET strategy. It is debated whether the application of CS might be extended even to cases not necessarily at risk of OHSS.

However, the weak points of CS mean it is not always convenient; it is undoubtedly more time-consuming and economically expensive for the IVF laboratory, and it may even represent an additional emotional burden for the patients, who may live with increasing anxiety the delay of their ET. 

For these reasons, even in patients at risk of OHSS, alternative strategies allowing fresh ET after the use of GnRH-analogue trigger have been conceived [6]. In fact, there is evidence that even very low levels of hCG might adequately support the luteal phase in GnRH-agonist triggered cycles, even without the addition of exogenous progesterone [20,35]. 

Over the last few years, the strategy of performing fresh ET and supplement the luteal phase with very low doses of hCG has been used in our IVF unit, as an alternative to traditional CS, in the case of non-PCO patients with ovarian reserve markers and a wide ovarian reserve (AMH > 2.5 ng/mL, AFC > 15), who were expected to deliver a high response to COS without the very high OHSS risk typical of patients with PCOS. When these patients obtained less than 20 oocytes at OPU, and the ultrasound (US) endometrial appearance at OPU was satisfactory (thickness > 7 mm and type 1 echogenicity), a fresh ET was scheduled, and the luteal phase was supplemented using 100 IU/d s.c. hCG. In contrast, when >19 oocytes were retrieved and/or the endometrium appeared thinner than 7 mm, or with type 2 or 3 US appearance, all blastocysts were vitrified, and ET was postponed in a subsequent, natural cycle with suitable endometrial characteristics.

The main finding of the present retrospective investigation is that the CS strategy allowed the obtaining of higher IR, CPR, and LBR, considering only the first ET than the strategy including fresh ET and hCG supplementation. This suggests that a very low daily dose of hCG, although overall safe with respect to the risk of OHSS, is probably inadequate to compensate for the negative effect of the trigger GnRH-agonist bolus on the luteal function. 

Indeed, women in the CS group had significantly higher AMH and AFC and, as expected, a better ovarian responsiveness to COS, more oocytes retrieved, and more available blastocysts. It is well known that there is a strong relationship between the number of oocytes and the LBR in a fresh IVF cycle, with optimal outcomes with between 15–20 eggs [36]. However, even normalizing for ovarian responsiveness through stratification in three subgroups of patients with a comparable number of retrieved oocytes and similar basal “a priori” characteristics, the analyses showed a better performance of the CS strategy, especially evident among patients retrieving >15 oocytes.

Notably, in the FRESH group, the LBR/ET was lower in the subgroup with 16–19 retrieved oocytes than in the two subgroups with fewer oocytes (*p* < 0.01), suggesting that when peak estradiol levels are higher, the inadequacy of hCG supplementation in restoring endometrial receptivity is probably more marked [37]. 

Conversely, the LBR/ET in the CS group showed an opposite trend, steadily increasing with the number of retrieved oocytes, an observation that confirms the association between ovarian responsiveness and the likelihood of getting a live birth [36]. 

As for safety issues, the strategy of performing a fresh ET with very low dose hCG supplementation was associated with an overall rate of OHSS (2.2% moderate plus severe OHSS rate), which compares favorably with the incidence of 3–6% commonly reported overall for IVF cycles [38]. However, it is questionable whether this rate of OHSS could still be acceptable, as the elimination of OHSS represents one of the main current goals of IVF treatments. 

## 4. Materials and Methods

The study was designed as a retrospective, real-life cohort study and was performed at the IVF Unit of Sant’Anna University Hospital after obtaining the approval of the Institutional Review Board, in accordance with the Helsinki Committee requirements (n. 0040486, 23/04/2020).

Among 2312 women undergoing IVF in the years 2016–2019, 434 were classified as expected high responders, according to their ovarian reserve markers (AMH > 2.5 ng/mL and antral follicle count (AFC) > 15). Of those women, 298 had regular menstrual cycles, no signs of hyperandrogenism, and no PCO appearance of the ovaries at transvaginal US; they were included in the study as non-PCO expected high responders. 

The remaining 136 women had polycystic ovary syndrome (PCO) according to ESHRE guidelines [39] and were excluded from the study. PCO patients, in fact, are known to represent a specific population that is at very high risk of developing severe OHSS, and according to our protocols, CS is mandatory. 

Other 58 women with multifollicular non-PCO ovaries were excluded from the study after OPU because >19 oocytes were retrieved, and, again, our protocols forced us to perform CS due to the well-known, direct relationship between the number of retrieved oocytes and the risk of severe OHSS. 

Therefore, finally, we compared 133 non-PCO high responders receiving fresh ET plus luteal phase hCG supplementation (FRESH group) vs. 107 comparable patients receiving cycle segmentation (CS group).

COS, OPU, and IVF or ICSI were performed as described in Appendix A. Both groups received a single subcutaneous injection of 0.2 mg Triptorelin Acetate (Decapetyl, Ferring, Germany) as an ovulation trigger. Embryos were assessed morphologically on day 2 using the 1–10 point scale score by Holte et al. [40], and then again on day 5 according to The Istanbul Consensus Workshop [41]. Embryos that progressed to the expanded blastocyst stage on day 5 (score 3) showed compacted inner cell mass (score 1), and cohesive trophoectoderm (score 1 or 2) were selected for the first transfer in both groups. The ET was performed as previously described [42]. 

The decision on whether patients would receive fresh ET or CS was taken during OPU and was based on the endometrial US appearance; when the endometrial thickness was <7 mm and/or its appearance was not trilinear (so it was type 2 or 3), CS was performed. In contrast, when the endometrial thickness was ≥7 mm, with a trilinear (type 1) appearance, fresh ET was chosen.

In the FRESH group, women received a single blastocyst fresh ET on day 5. Starting the day after OPU, 100 IU/day of hCG (Gonasi HP, Ibsa, Lugano, Switzerland) were administered subcutaneously for 14 days. 

In the CS group, a standard vitrification protocol was used to freeze all blastocysts. ET was performed in one of the following menstrual cycles, monitoring spontaneous ovulation by urinary LH-detection kit (Clearblue^®^ Ovulation Test, Swiss Precision Diagnostics, Switzerland). ET with one thawed blastocyst was performed only whether endometrial thickness reached at least 7 mm, with an endometrial US type 1 appearance the day after urinary LH peak. The luteal phase was supplemented with 180 mg/day intra-vaginal natural progesterone (Crinone 8, Merck, Germany), starting 48 h after the LH peak and continuing for 14 days. 

In case of a positive pregnancy test, transvaginal ultrasound (TV-US) examination was scheduled two weeks later.

The implantation rate (IR) was calculated, taking into account also the biochemical pregnancies. The clinical pregnancy rate (CPR) was calculated considering only pregnancies that were confirmed at TV-US (presence of a gestational sac) two weeks after the positive pregnancy test. Live birth rate (LBR) was calculated with live birth defined as a live-born infant after >24 weeks of gestation. 

The number of retrieved oocytes is a well-known factor affecting the LBR [36]; to minimize the impact of this variable, a sub-analysis was performed, in which FRESH and CS groups were compared after stratification for the number of retrieved oocytes into three subgroups (8–11, 12–15, or 16–19). 

Safety was evaluated considering the incidence of OHSS, whose severity was assessed as previously described [43]. Statistical comparison between the two study groups was performed using the GraphPad Prism V7 software package and Stata16; the Student’s *t*-test or the non-parametric Wilcoxon rank-sum test were used, as appropriate, for continuous variables (shown as mean ± SD and/or median and interquartile range (IQR)), whereas the Chi squared or Fisher’s tests were used for categorical variables (shown as percentages and absolute frequencies). Multivariable analysis models were then used to investigate the independent effects of the study group (CS vs. FRESH) on LBR in the three subgroups of women, stratified according to the number of retrieved oocytes. Logistic regression models were fit using LBR as dependent variable and study group, with AMH and estradiol levels as independent variables. All statistical tests were two-sided, and a *p*-value of 0.05 was considered the significance threshold. 

## 5. Conclusions

In conclusion, to the best of our knowledge, this is the largest study comparing IVF outcomes in non-PCO high responders submitted to COS with GnRH-antagonist protocol, GnRH-agonist trigger, and either CS or fresh ET with hCG luteal phase support. 

With the limitation of its retrospective nature, the present study shows that the strategy of CS is associated with higher IR, CPR, and LBR than the alternative strategy, including fresh ET plus repeated very low daily dose of hCG. 

If confirmed using a larger sample size and in a randomized controlled trial, the present findings would help to orient clinical decisions, with the final aim of improving IVF outcomes.

## Figures and Tables

**Table 1:** Clinical baseline characteristics of the patients receiving fresh embryo transfer (ET) plus human chorionic gonadotropin (hCG) luteal supplementation (FRESH group) vs. those undergoing cycle segmentation with freezing of all embryos and delayed ET (CS group). Data are expressed as mean ± standard deviation.

Clinical Baseline Characteristics	FRESH (*n* = 133)	CS (*n* = 107)	*p*-Value
Age (years)	34.7 ± 4.1	34.8 ± 4.1	0.80
BMI (kg/cm^2^)	22.6 ± 3.7	22.8 ± 4.6	0.79
Basal AMH (ng/mL)	5.3 ± 2.9	7.4 ± 5.6	<0.01
Basal (day 3) FSH (UI/L)	6.4 ± 1.4	6.4 ± 1.7	0.91
Basal (day 3) LH (UI/L)	6.6 ± 3.1	7.3 ± 3.5	0.14
Antral follicle count (AFC)	19.9 ± 6.8	26.9 ± 10.1	<0.01

LH: luteinizing hormone.

**Table 2:** In vitro fertilization (IVF) outcomes of patients receiving fresh ET plus hCG luteal supplementation (FRESH group) vs. those undergoing cycle segmentation with freezing of all embryos and delayed ET (CS group). Data are expressed as mean ± standard deviation and median (IQR), if appropriate, for quantitative variables and percentages for qualitative variables. * Endometrial thickness in the FRESH group was measured during oocyte pick up (OPU), whereas in the CS group, it was measured the day after LH urinary peak during the spontaneous cycle in which ET with thawed embryo was performed. ** In both groups, only the first ET was considered. OHSS: ovarian hyperstimulation syndrome.

IVF Cycle Outcomes	FRESH (*n* = 133)	CS (*n* = 107)	*p*-Value
Mean daily FSH dose (UI) Median (IQR)	169.4 ± 38.9 150 (150–194)	164.8 ± 40.1 150 (150–187.5)	0.40
Total FSH dose (UI) Median (IQR)	1774 ± 557.8 1650 (1350–2000)	1659.9 ± 519.4 1600 (1350–1948)	0.21
Estradiol at ovulation trigger (pg/mL) Median (IQR)	2332.1 ± 1026.2 2142 (1554–2966)	3573.8 ± 2163.2 3203 (2265–4363)	<0.01
Endometrial thickness (mm) *	10.3 ± 1.7	10 ± 2	0.14
Retrieved oocytes (*n*) Median (IQR)	12.6 ± 3.1 13 (10–14)	14.2 ± 3.4 15 (11–17)	<0.01
Mature (MII) oocytes (*n*)	10.3 ± 3.1	11 ± 3.6	0.11
Fertilization rate (%) Median (IQR)	63.7 ± 19.9 66.7 (50–77.8)	70.8 ± 20.9 72.2 (55.5–90)	<0.01
Mean embryo score (*n*) Median (IQR)	7 ± 1.4 7.1 (6.1–7.9)	7.2 ± 1.5 7.1 (6.4–8.2)	0.55
Blastocysts (*n*) Median (IQR)	3.3 ± 2.6 3 (1–5)	4.5 ± 2.7 4 (2–6)	<0.01
Implantation rate (%) **	27.8	42.9	<0.05
Clinical pregnancy rate/first ET% **	26.3	40.2	<0.05
Live birth rate/first ET% **	19.5	32.7	<0.05
Mild OHSS%	12	12.1	0.98
Moderate OHSS%	1.5	0	-
Severe OHSS%	0.8	0	-

**Table 3:** Clinical baseline characteristics and IVF outcome of the patients receiving fresh ET plus hCG luteal supplementation (FRESH group) vs. those undergoing cycle segmentation with freezing of all embryos and delayed ET (CS group). Women were stratified according to the number of retrieved oocytes in order to normalize for the ovarian response to COS: A = 8–11 retrieved oocytes; B = 12–15 retrieved oocytes; C = 16–19 retrieved oocytes. Data are expressed as mean ± standard deviation or median (IQR), as appropriate, if not otherwise stated. ** In both groups, only the first ET was considered.

**A (8–11 Retrieved Oocytes)**	**FRESH** **(*n* = 52)**	**CS** **(*n* = 30)**	***p*-Value**
Age (years)	34.8 ± 4.3	34.9 ± 4.2	0.94
BMI (kg/cm^2^)	23.1 ± 3.4	23.6 ± 6.2	0.63
Basal AMH (ng/mL)	4.15 (2.8–6)	5.2 (3.3–6.5)	0.13
Estradiol at ovulation trigger (pg/mL)	2070 (1529–2920.5)	2485.5 (1746–4268)	0.06
Positive hCG test % (*n*)	25 (13)	30 (9)	0.62
Live birth rate/first ET% (*n*) **	21.2 (11)	23.3 (7)	1.00
**B (12–15 Retrieved Oocytes)**	**FRESH** **(*n* = 60)**	**CS** **(*n* = 32)**	
Age (years)	34.7 ± 4.1	34.4 ± 3.7	0.73
BMI (kg/cm^2^)	22.1 ± 3.3	22.5 ± 3.4	0.63
Basal AMH (ng/mL)	5.45 (3.4–6.3)	6.3 (3.8–8.7)	0.07
Estradiol at ovulation trigger (pg/mL)	2284.5 (1609.5–3090)	3254.5 (2342–4311)	<0.01
Positive hCG test % (*n*)	31.7 (19)	40.6 (13)	0.49
Live birth rate/first ET % (*n*) **	21.7 (13)	28.1 (9)	0.61
**C (16–19 Retrieved Oocytes)**	**Fresh** **(*n* = 21)**	**CS** **(*n* = 45)**	
Age (years)	34.3 ± 3.8	35.1 ± 4.3	0.48
BMI (kg/cm^2^)	22.8 ± 5.5	22.4 ± 4.1	0.76
Basal AMH (ng/mL)	5.7 (4.3–6.5)	6.9 (4.4–9.8)	0.07
Estradiol at ovulation trigger (pg/mL)	1918 (1583–3024)	3345 (2445–4363)	<0.01
Positive hCG test % (*n*)	33.3 (7)	51.1 (23)	0.19
Live birth rate/first ET% (*n*) **	9.5 (2)	42.2 (19)	0.01

## Data Availability

All data are available under request to the authors

## Appendix A

All the enrolled non-PCO expected high responders underwent COS with the GnRH-antagonist regimen, using recombinant FSH (Gonal F, Merck, Darmstadt, Germany or Puregon, MSD, Readington, NJ, USA). The FSH starting dose ranged from 100 to 150 IU according to age and BMI and was then adjusted from stimulation day 5–7 according to the ovarian response at the first checkpoint, during which transvaginal US (TV-US) and serum estradiol (E2) measurement were performed. From stimulation day 5, a daily injection (0.25 mg/d s.c) of GnRH-antagonist (Cetrorelix Merck, Darmstadt, Germany or Orgalutran, MSD, Readington, NJ, USA) was added. Follicular growth was monitored by TV-US and serum E2 every 2–3 days, and when at least two follicles reached 18 mm mean diameter, with appropriate E2 circulating levels, ovulation was triggered by a single subcutaneous injection of 0.2 mg Triptorelin Acetate (Decapetyl, Ferring, Saint-Prex, Switzerland). Ovulation trigger was performed in the same way in the FRESH and CS groups. OPU was performed approximately 36 h after Triptorelin Acetate injection by US-guided aspiration under local anesthesia (paracervical block). Follicular fluid was immediately observed under a stereomicroscope; cumulus-oocyte complexes (COCs) were washed in buffered medium (Flushing medium, Cook Ltd., Limerick, Ireland), and oocytes were inseminated within 4 h from OPU using either conventional IVF or ICSI, according to the quality of the semen sample. Normal fertilization was confirmed when the presence of two pronuclei (2PN) and the extrusion of the second polar body were observed 16–18 h after oocyte insemination. Zygotes were placed in 4-well dishes (Thermo Ficher Scientific, Waltham, MA, USA) and cultured in pre-equilibrated cleavage medium (Cook Ltd., Limerick, Ireland) overlain with mineral oil (Culture Oil, Cook Ltd., Limerick, Ireland).
